# Post-transcriptional regulation of gene expression in bacterial pathogens by toxin-antitoxin systems

**DOI:** 10.3389/fcimb.2014.00006

**Published:** 2014-01-29

**Authors:** Ralph Bertram, Christopher F. Schuster

**Affiliations:** Department of Microbial Genetics, Faculty of Science, Interfaculty Institute of Microbiology and Infection Medicine Tübingen (IMIT), University of TübingenTübingen, Germany

**Keywords:** toxin-antitoxin system, TA system, RNase, gene regulation, translation inhibition, pathogenicity, review

## Abstract

Toxin-antitoxin (TA) systems are small genetic elements ubiquitous in prokaryotic genomes that encode toxic proteins targeting various vital cellular functions. Typically, toxin activity is controlled by adjacently encoded protein or RNA antitoxins and unleashed as a consequence of genetic fluctuations or stressful conditions. Whereas some TA systems interfere with replication or cell wall synthesis, most of them influence transcriptional and post-transcriptional gene regulation. Antitoxin proteins often act as DNA binding transcriptional regulators and many TA toxins exhibit endoribonuclease activity to selectively degrade different RNA species and thus alter gene expression patterns. Some TA RNases cleave tRNA, tmRNAs or rRNAs, whereas most commonly mRNAs either in association with the ribosome or as free transcripts, are targeted. Examples are provided on how TA toxins differentially shape gene expression in bacterial pathogens by creating specialized ribosomes or by altering the transcriptome and how this may be tied in the control of pathogenicity factors.

## About toxin-antitoxin systems

Toxin-antitoxin (TA) systems are small and frequently bicistronic elements that are widely distributed throughout bacterial and archaeal genomes. They generally encode a low molecular weight protein which interferes with vital cellular functions and another protein or RNA molecule that keeps the toxin activity in check. The first TA loci have been characterized as plasmid addiction systems on *E. coli* episomes. There, cells which suffered stochastic plasmid loss in the course of cell division were found to be eradicated by a stable TA toxin whose less stable cognate antitoxin was no longer produced. For example, the *hok-sok* system ensures faithful partitioning of the plasmid R1 via the membrane pore forming Hok toxin (Gerdes et al., 1986), whereas the F-plasmid is stabilized in a population through the gyrase inhibitor activity of the *ccdAB* locus (Ogura and Hiraga, 1983; Jaffé et al., 1985). Dozens of further TA systems have been identified since (Pandey and Gerdes, 2005; Makarova et al., 2009; Fozo et al., 2010; Blower et al., 2012; Aakre et al., 2013) and according to the mode of action and chemical nature of the antitoxin, they to date fall into at least five types, as reviewed recently (Schuster and Bertram, 2013). Toxin activity of type I TA systems is post-transcriptionally controlled by a small non-coding RNA antitoxin, whereas in Type II TA systems, the toxin protein is inactivated by direct interaction with the antitoxin protein. More recently, type III, IV, and V TA systems have been described, albeit represented by only few validated examples (Fineran et al., 2009; Masuda et al., 2012; Wang et al., 2012). Whereas type IV and V TA systems are genetically similar to type II systems, type III TA toxin proteins are bound by non-coding RNA antitoxins in the inactive state. Only very recently a new type of TA system was identified which features an unstable toxin protein that is degraded by Clp in association with the cognate antitoxin (Aakre et al., 2013). According to this novel mode of activity control, it might be justified to define it as a novel type VI TA system (Markovski and Wickner, 2013).

**Figure 1:**
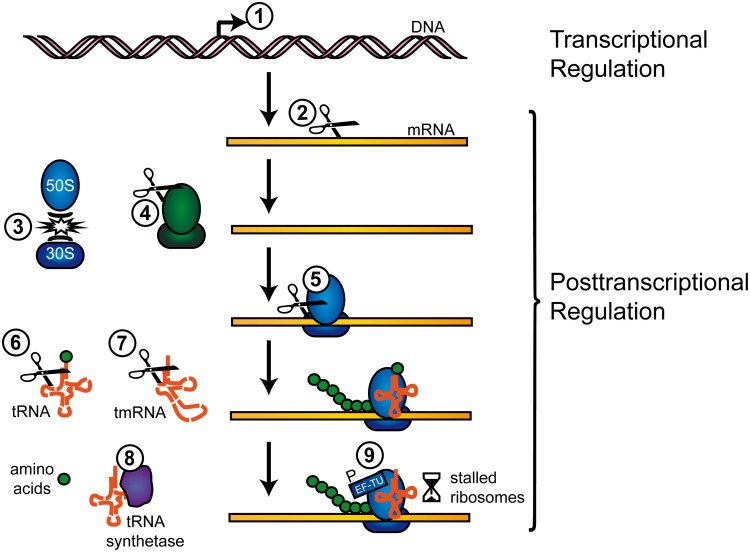
**Regulation of transcription and translation by TA systems.** TA systems can regulate gene expression on a transcriptional and post-transcriptional level, as follows (examples in parentheses): **(1)** Canonical transcriptional gene regulation by DNA binding (MqsR). **(2)** Cleavage of free mRNA transcripts (MazF, ChpBK, PemK, HicA). **(3)** Inhibition of ribosome association (RatA, MazF). **(4)** Cleavage of rRNAs (MazF, VapC). **(5)** Cleavage of ribosome associated mRNAs (RelE, YoeB). **(6)** Cleavage of tRNAs (VapC). **(7)** Cleavage of tmRNAs (HicA, RelE). **(8)** Inhibition of tRNA synthetase (HipA). **(9)** Phosphorylation of EF-Tu (Doc). Further information on the mechanisms is available in the respective sections.

Generally, TA toxin activity is unleashed by an imbalance in favor of free toxin molecules, generated either by stochastic fluctuations in the TA systems' gene expression or cellular stress (Christensen et al., 2003; Vogel et al., 2004; Dörr et al., 2010; Maisonneuve et al., 2013). Antitoxin proteins of type II TA systems are frequently proteolyzed by Clp or Lon factors (Brzozowska and Zielenkiewicz, 2013) and hence toxin-antitoxin stoichiometries are shifted. The abundance of TA systems in prokaryotic chromosomes has sparked vivid discussions about their physiological roles beyond plasmid maintenance, including functions in programmed cell death, bacterial growth impediment, defense against foreign genetic material, or (fine) tuning of the physiological activity to shift cells into dormant, drug-tolerant states (Magnuson, 2007; Lewis, 2010; Gerdes and Maisonneuve, 2012). TA toxins target a variety of vital cellular structures and functions within bacteria such as membrane integrity, replication, cell wall synthesis, ribosome assembly, and translation-factors, with RNA cleavage as the most prevalent mode of action (Schuster and Bertram, 2013). Type II TA systems have been grouped into fuzzy numbers of families and superfamilies (Gerdes et al., 2005; Leplae et al., 2011) and TA RNases can further be distinguished according to their ribosome-dependency (Yamaguchi and Inouye, 2011). Recent reviews highlight the mechanisms of RNA maturation and degradation and the involvement of TA encoded RNA interferases (Yamaguchi and Inouye, 2009; Rochat et al., 2013). We here provide examples on how TA systems impact the flow of genetic information in pathogenic bacteria, with a focus on post-transcriptional regulation via TA toxin RNase activity (summarized in Figure 1).

## VapC homologs target a multitude of substrates in different bacteria

Type II TA systems of the VapBC type are highly abundant in prokaryotes and frequently, multiple paralogs of them are found in bacterial genomes (Pandey and Gerdes, 2005; Jørgensen et al., 2009). In Mycobacteria, the number of identified VapBC type TA systems ranges between one in *M. smegmatis* to more than 40 in the pathogen *M. tuberculosis* (Pandey and Gerdes, 2005). A hallmark of the VapC toxins is the PIN domain, which is typically associated with Mg^2+^ dependent ribonuclease activity (Arcus et al., 2004). Sharp et al. (2012) determined the ACGC or AC[AU]GC motifs of mRNAs as targets of the VapC-mt4 protein of *M. tuberculosis* and reported inhibition of translation mostly by binding to ssRNA instead of cleaving it. Experiments with the VapC20 (Rv2549c) homolog of *M. tuberculosis in vitro* and in *E. coli* have shown cleavage of the 23S ribosomal RNA between G and A in a prominent loop region (Winther et al., 2013). By contrast, purified VapC of *M. smegmatis* exhibits RNase activity preferentially at AUAU and AUAA sites (McKenzie et al., 2012). Microarray studies demonstrated differential expression of almost 3% of the *M. smegmatis* genome upon overexpression of the toxin with a striking downregulation of carbohydrate metabolism genes. The identification of VapC as a post-transcriptional regulator of glycerol consumption in mycobacteria highlights the influence of TA systems in bacterial metabolism. In addition, Winther and Gerdes (2009) were able to link induction of *vapC* expression in *Salmonella enterica* serovar Typhimurium to the stringent response. In contrast to analyzed homologs from Mycobacteria, this VapC protein and the one encoded on the *Shigella flexneri* 2a virulence plasmid pMYSH6000 exclusively target tRNA^fMet^ by cleaving within the anticodon stem-loop. Together with the RelE-type toxin YoeB (see below), the concomitant stimulation of translation initiation at elongator codons in lieu of canonical start codons may globally affect the cellular translation program (Winther and Gerdes, 2011).

## The hicAB TA homologs

Features like induction by the stringent response and cleavage of tmRNA, required to rescue stalled ribosomes (Keiler et al., 1996), are shared by the *E. coli hicAB* TA system, which can also be activated during carbon starvation by a Lon protease dependent process. The HicA toxin furthermore cleaves selected mRNAs such as transcripts of *dksA*, and *rpoD* encoding regulators or subunits of the RNA polymerase or *ompA*, an outer membrane protein (Jørgensen et al., 2009). An orthologous system from the opportunistic pathogen *Acinetobacter baumannii* also targets tmRNA and a set of mRNAs when expressed in *E. coli* (Jurėnaitė et al., 2013). To date, it is unclear if and how the *hicAB* locus regulates differential gene expression in the host-cell, but it is likely that some genes are post-transcriptionally influenced by HicA-dependent cleavage.

## MazEF, a jack-of-all-trades system

*mazEF* belongs to the best characterized TA systems and encodes a MazF toxin and a MazE antitoxin protein. MazF homologs have been identified in a large number of bacteria (Mittenhuber, 1999) and archaea, with the *E. coli* system representing the far most extensively studied one (Aizenman et al., 1996). *E. coli* MazF is an ACA sequence specific endoribonuclease, which upon induction cleaves the bulk of all cellular mRNAs (Zhang et al., 2003). Accordingly, as much as 90% of the encoded proteins are no longer produced, leading to growth impediment (Yamaguchi and Inouye, 2009). While it had previously been assumed that ribosomal RNAs and tRNAs are protected from MazF cleavage by ribosomal proteins and secondary structures (Zhang et al., 2005a). Vesper et al. (2011) demonstrated that 16S rRNAs are processed by *E. coli* MazF, presumably in response to stressful conditions. Thereby the anti-Shine-Dalgarno (anti-SD) sequence of the 16S rRNA is removed, yielding so called “stress ribosomes”. These are capable of translating a set of leaderless mRNAs with clipped SD sequences as another result of sequence specific MazF activity. This orthogonal translation system produces about 50 proteins which are associated with population heterogeneity in *E. coli* cultures that leaves only few survivors behind (Amitai et al., 2009). The controversial role of the *mazEF* system is reflected by two opposite camps. One suggests an involvement in programmed cell death (Aizenman et al., 1996; Amitai et al., 2004; Engelberg-Kulka et al., 2006), whereas the other provides evidence for the modulation of physiological activities for a defined time-frame only (Pedersen et al., 2002; Gerdes and Maisonneuve, 2012). The proficiency of *E. coli* MazF and a homologous TA toxin ChpBK, which exhibits less stringent ACA specificity (Zhang et al., 2005b), appear to be enhanced by a pentapeptide called extracellular death factor (EDF) (Kolodkin-Gal et al., 2007; Belitsky et al., 2011). Functional analogs thereof are also produced by *P. aeruginosa* and *B. subtilis* (Kumar et al., 2013). *Mycobacterium tuberculosis* bears at least seven *mazEF* operons, four of which were validated to encode functional mRNA interferases, targeting different three- or five-base consensus sequences (Zhu et al., 2006, 2008). These MazF proteins were proposed to alter gene expression through selective RNA degradation by each paralog addressing different targets. These also include RNA species beyond mRNA, as in case of MazF-mt6. It cleaves 23S rRNA of *M. tuberculosis* in a bulge region that is part of an association interface between the 30S and the 50S subunits. This leads to an mRNA independent global shut-down of translation activity (Schifano et al., 2013). Interestingly, MazF-mt7 (Rv1495) can physically interact with the *M. tuberculosis* DNA topoisomerase I (MtbTopA), which inhibits nucleic acid cleavage activity of both enzymes. This interaction exemplifies an additional regulatory function of a TA interferase beyond ribonuclease activity (Huang and He, 2010). Heterologous expression of MazF-mt7 in *M. smegmatis* causes growth cessation, which, together with findings on the MazF-mt6 system, provides further indications for the involvement of TA systems in mycobacterial long-term dormancy. Among the Gram positive pathogens, *mazEF* systems were also identified in *Clostridium difficile*, *Streptococcus mutans*, and *Staphylococcus aureus* (Fu et al., 2009; Zhu et al., 2009; Syed et al., 2011; Rothenbacher et al., 2012). Relative abundances of target sites may serve as gene specific indicators for cleavage sensitivity, direct evidence is mostly lacking and secondary structures and RNA associated factors can influence accessibility. This has striking implications for post-transcriptional regulation of factors associated with host-pathogen interactions. Recently, a *pemIK* system generally related to *mazEF* (Zhang et al., 2004), was identified on two staphylococcal plasmids and in the chromosomes of other *Staphylococcus* species (Bukowski et al., 2013). Whereas PemIK_Sa_ fulfils the classical TA systems' role of ensuring plasmid propagation, its toxin component PemK_Sa_, an UAUU specific endoribonuclease, has also been speculated to post-transcriptionally control chromosomally located *S. aureus* genes. Transcripts biased for a low number of UAUU stretches include ORFs of various virulence factors, whereas numerous transporter genes harbor more target sequences than statistically predicted. The finding that the transcript encoding the PemI_Sa_ antitoxin is resistant to PemK_Sa_ cleavage led the authors to propose a mechanism for reinstating TA homeostasis after stressful conditions (Bukowski et al., 2013).

## Ribosome dependent RNases of the RelE family

The RelE toxins of the *relBE* TA family interact with the ribosome and cleave mRNAs at the ribosomal A-site (Pedersen et al., 2003). *E. coli* RelE preferentially targets the trimeric RNA motifs UAG, UCG, and CAG. Interestingly, tmRNA can also be a substrate of RelE, which suggests a role in stress regulation (Christensen and Gerdes, 2003). YafQ toxins, which are structurally similar to RelE family RNases, associate with the 50S ribosomal subunit and specifically restrict selected mRNAs at AAA[AG] consensus sequences. Since the AAA lysin codon is particularly overrepresented at codon +2 in secretory proteins (Zalucki et al., 2007), RelE activity may arrest their translation (Prysak et al., 2009). More recently, also *in vivo* cleavage of the transcripts *lpp* (lipoprotein), *acpP* (acyl carrier protein) and *hns* (DNA condensing and supercoiling protein) at AAG, GAA, and ACA was demonstrated (Armalytė et al., 2012). Likewise, the *E. coli* YoeB toxin binds to the 50S ribosomal subunit and cleaves the *lpp* and *ompA* (porin) mRNA *in vivo*, three nucleotides downstream of the start codon (Zhang and Inouye, 2009). The YefM-YoeB system promotes colonization of the bladder by uropathogenic *E. coli* (Norton and Mulvey, 2012) and is also found in the chromosome of Gram positive pathogens such as *Staphylococcus aureus*, *Streptococcus pneumoniae*, and *Mycobacterium tuberculosis* (Cherny and Gazit, 2004; Nieto et al., 2007; Yoshizumi et al., 2009). A homologous TA system, termed *axe-txe*, has been identified on *Enterococcus* plasmids before (Grady and Hayes, 2003; Halvorsen et al., 2011) and Txe was shown to cleave *lpp* mRNA at the first base after an AUG start codon when expressed in *E. coli*. Apart from a number of *in vivo* and *in vitro* assays which demonstrate the ribosome dependency for these TA toxins, a holistic picture of which genes are affected under specific conditions is yet to be painted for most of these systems. An exception is the RelE family toxin HigB from the *higBA* TA system, which also cleaves RNA in association with the ribosome (Hurley and Woychik, 2009). Schuessler et al. (2013) analyzed an *M. tuberculosis* strain overexpressing *higB* by comparative RNAseq in order to define the entire set of RNAs targeted by the toxin. The relative abundances of tmRNA and of 32 different mRNAs was decreased, of which many are controlled by regulators associated with iron acquisition and stress response (Keiler et al., 1996). It should be noted that although these approaches reveal the complete set of directly and indirectly regulated genes, they do not disclose the targeted nucleotide sequence of the respective loci.

## MqsRA modulates *E. coli* stress response

Whereas many TA systems have been solely characterized *in vitro* or by heterologous expression *in vivo*, a distinct physiological role of the type II TA system MqsRA (Yamaguchi et al., 2009) and the modulation of the *E. coli* stress response has been established (Wang et al., 2011). Apart from MsqA functioning as a transcriptional stress regulator, the MqsR toxin acts as a sequence-specific RNA-interferase. *In vivo* primer extensions revealed cleavage of three different native *E. coli* transcripts at the GCU triplet upon artificial induction of MqsR. All of the GCU sequences in *ompF* mRNA were cleaved without exception although mRNAs form secondary and tertiary structures and can associate with proteins. It was speculated that the MqsRA system could also have important implications for uropathogenic strains switching from a motile non-adhesive state in the urine to a recalcitrant biofilm or other types of non-motile bacterial communities (Hadjifrangiskou et al., 2011).

As shown recently, MqsRA also controls another TA system, termed *ghoST* (Wang et al., 2013). The small GhoT toxin protein inserts into the membrane resulting in lysed (“ghosts”) or drug tolerant persister cells (Cheng et al., 2013). Notably, *ghoT* mRNA lacks the primary MqsR target site GCU (Yamaguchi et al., 2009), but instead is specifically cleaved by the GhoS antitoxin. GhoS in turn targets another 20 mRNAs involved in nucleotide precursor anabolism (Wang et al., 2012). Cleavage of toxin mRNA has so far exclusively been described for GhoST, which represents a type V TA system member with extraordinary activities of self-control.

## Additional modes of post-transcriptional regulation

Apart from gene regulation based upon RNA degradation, a number of TA systems exert post-transcriptional regulation by targeting downstream processes.

Like mycobacterial MazF-mt6 (see above), the RatA toxin, encoded by the *ratAB* locus from *E. coli*, inhibits the association of the ribosomal subunits. Although already assembled ribosomes are unaffected, synthesis of new proteins is impeded (Zhang and Inouye, 2011).

The PhD/Doc system blocks general translation by binding to the 30S ribosome subunit and thus inhibiting translation elongation (Liu et al., 2008). As found recently, the toxicity of the Doc-type protein Fic is mediated by phosphorylation of the elongation factor EF-Tu, which in turn prevents binding to aminoacetylated tRNAs (Castro-Roa et al., 2013).

The prominent *E. coli hipAB* TA system, which is involved in a high persister phenotype, was thought to phosphorylate EF-Tu to nonspecifically inhibit overall translation (Schumacher et al., 2009). Newest results, however, indicate that translation inhibition is caused by impeding the glutamyl-tRNA synthetase, thus leading to an accumulation of uncharged tRNAs and therefore translation arrest (Germain et al., 2013). Although these systems are presumably non-selective regulators due to the unspecific shutdown of translation in general, they may regulate translation of total transcriptomes in stress related situations.

## Closing remarks

Gone are the days when TA systems were considered to solely act as plasmid stabilizers. Instead, a multitude of roles for these genetic elements in the modulation of bacterial physiology has emerged. It became evident that TA systems target numerous intracellular structures and processes in prokaryotes among which the modulation of translational cessation seems to be of prime importance. First approaches have been taken to decipher the entirety of transcripts (including small regulatory RNAs) affected by TA RNases throughout different bacteria (Kim et al., 2010; Schuessler et al., 2013). In this regard, secondary structures of RNA molecules, factors interacting with RNAs such as Hfq or other RNA chaperones (Vogel and Luisi, 2011), as well as activity modulators of TA system RNases, including peptides (Kolodkin-Gal et al., 2007; Kumar et al., 2013) or hierarchically organization of TA systems may need to be considered in greater detail (Winther and Gerdes, 2009; Wang et al., 2012). Most importantly, to elucidate regulatory networks, TA systems must be studied in their cellular and environmental context, as heterologous expression in *E. coli* and activities observed *in vitro* most likely reflect only part of the extent of regulation. Numerous clinical isolates of *S. aureus* and *P. aeruginosa* have been demonstrated to harbor and express TA systems (Williams et al., 2011), which permits refined *in vivo* analyses without the need for spurious noise due to artificial induction. Another recently discovered antitoxin-like protein in an enterohaemorrhagic *E. coli* phage can directly undermine the native TA systems and thus nicely underlines the importance of homologous *in vivo* experiments (Otsuka and Yonesaki, 2012).

Insights into extended roles of type II antitoxins are emerging. Besides controlling toxin activity by protein-protein interaction and transcriptional autoregulation, some antitoxins have been proven to additionally act as transcription factors controlling other regulons (Kim et al., 2010; Lin et al., 2013). Recent findings underscore the hypothesis that pathogenic bacteria contain higher numbers of TA systems than non-pathogenic relatives (Georgiades and Raoult, 2011). A recent publication furthermore showed a direct effect of a newly discovered *Salmonella* TA system (*sehAB*) and virulence in a mouse model (De la Cruz et al., 2013). Taken together, TA systems provide interesting targets for antibacterial strategies (Williams and Hergenrother, 2012), which clearly merits further research in this dynamic field.

### Conflict of interest statement

The authors declare that the research was conducted in the absence of any commercial or financial relationships that could be construed as a potential conflict of interest.
